# Nursing Personnel Planning for Rural Hospitals in Burdwan District, West Bengal, India, Using Workload Indicators of Staffing Needs

**Published:** 2014-12

**Authors:** Swapnil Shivam, Rabindra Nath Roy, Samir Dasgupta, Krishna Das Bhattacharyya, Raghu Nath Misra, Sima Roy, Indranil Saha

**Affiliations:** ^1^Community Medicine, Burdwan Medical College and Hospital, Burdwan 713104, West Bengal, India; ^2^Community Medicine, Institute of Postgraduate Medical Education and Research, Kolkata 700 020, West Bengal, India

**Keywords:** Activity standards, Staffing needs, WISN, Workload, Workforce projection

## Abstract

Lack of appropriate human resources planning is an important factor in the inefficient use of the public health facilities. Workforce projections can be improved by using objective methods of staffing needs based on the workload and actual work undertaken by workers, a guideline developed by Peter J. Shipp in collaboration with WHO—Workload Indicators of Staffing Need (WISN). A cross-sectional study was carried out to estimate the nursing stuff requirement for the rural hospitals and provide a quantitative description of imbalances, if there is any, in the allocation at the district level during 2011. The average WISN turns out to be 0.35 for entire district, which means only 35% of the required nurses is available or 65% understaffed. So, there is an urgent need for more allocations and deployment of staff so that workload can be tackled and evenly distributed among all nursing personnel.

## INTRODUCTION

Continuing and worsening shortages of nurse nationally and internationally have been a matter of concern (1). Shortage of health workers in low-income countries is a major obstacle to attaining the Millennium Development Goals (2). In addition to the general shortage of health workers, inequalities in the distribution of health workers further add to this problem (3). Besides meeting the requirement, retention of nursing personal in service is a matter of concern to healthcare mangers. The workload is one of the most significant factors that affect the retention of nursing personnel in a service (1). The link between nursing workloads and quality of care, including patient safety is well-established. Besides the understaffed working station, high bed-occupancy rate also adversely affect the quality of service. A generally accepted standard of safe hospital occupancy is 85%; yet most hospitals worldwide have occupancy of around 100% or higher. The problem of overcrowding leads to high rates of hospital-acquired infections and unnecessary re-admission in hospital, compounded by high nursing workload, results in vicious circle of poor quality of care. Governments should commit to achieving safe staffing across the continuum of care to ensure quality service (4).

Presently, policy-makers in India are concerned about both efficiency and equity in the use of resources in the health sector. In the task of making estimation for the existing and projected manpower needs in the health sector, there are a number of problems, such as (a) highly-inadequate information system in health; (b) lack or laxity in registration systems and current stock; (c) inadequate information on supply; (d) lack of information on migration, death rates, and attrition on other grounds; and (e) methodological problems relating to estimation of manpower requirements (5). Efforts should be undertaken to make the projections of human resources more rational and realistic. In the absence of any objective method of determining staffing needs, some subjective types of staffing norm are currently used, mostly based on the population norms and institution-size. This does not take into consideration the regional variations in the utilization patterns. National health workforce strategies require reliable and timely information, rational system analysis, and a firm knowledge base for human resource planning. Thus, workforce projections can be improved greatly by using objective methods of staffing needs rather than the subjective staffing norms commonly used. There is neither sufficient nor reliable scientific evidence regarding the workload and equity in the distribution of nurses in the public healthcare system in India; workload is also not used in decision-making for deployment in nursing services. As workload is one of the most significant factors that affect the retention of nursing personnel in any service, it is of utmost importance to measuring nursing workload reliably to understand the relationship between workload, retention of nurses, and patient safety.

Peter J. Shipp, in collaboration with WHO, Geneva, has developed guidelines to determine Workload Indicators of Staffing Need (WISN) (6). The WISN Method determines staffing requirements for each category based on the workload of the facility. The present study was undertaken to estimate quantitatively the staffing need of nursing staff based on ‘activity standards’ and ‘workload’ (using Workload Indicators of Staffing Need guidelines) in all rural hospitals of Burdwan district, West Bengal, India, and to assess the imbalances, if any, in the deployment of nursing staff in those hospitals.

## MATERIALS AND METHODS

A descriptive cross-sectional study was carried out during April 2012 to March 2013 in Burdwan district in the state of West Bengal, India. Burdwan, a district of West Bengal (an eastern state of the country), with a population of 7,717,563 is the most densely-populated district (nearly 1,100/square km) (7) in West Bengal. The data for the study were collected in January 2012, which refer to the workload statistics of January 2011 to December 2011. The study population comprised all 6 rural hospitals in the district, along with the nursing staff working in these hospitals. For identifying different activities for patient-care services and average time required for each component of services, one of the rural hospitals had to be identified as standard institution as per WISN manual. By interviewing some key informants (district health officials), rural hospital in Manker had been identified as the “average and typical” one. For setting activity standard, i.e. the time it would take for a well-trained and well-motivated member of a particular staff category to perform an activity at acceptable professional standards, senior nursing staff members of rural hospital in Manker were interviewed. The tools for data collection were adopted and customized from the WISN manual. It was pretested in a rural hospital of Hooghly district, West Bengal, India. The present numbers of nurses (sanctioned, posted, on deputation, vacant) were obtained from the hospital stafflist for the month of January 2011.

### Basis of the WISN Method

The WISN Method is based on the work which is actually undertaken by health staff. Every health facility has its own pattern of workload, which may include inpatients, surgical operations, deliveries, outpatients, etc. Each type of workload calls for effort (i.e. time) from specific health staff category (Figure).

The basis of the WISN Method is activity standard, which is the time it would take for a well-trained and well-motivated member of a particular staff category to perform an activity at acceptable professional standards. In this study, the nursing personnel with substantial experience in their field having thorough knowledge about the nursing practice and administrative issues were interviewed to identify different activities and set working time for each component. There were, sometimes, differences in opinion regarding time requirement; in such situation consensus were reached taking average of opinions of three experts. According to Peter J. Shipp, the author of the original WISN manual, experts are “expected to bring to bear professional expertise and recent working experience.” We also found it difficult to group different activities for setting activity standard as there were a lot of subtasks in each of the components of activity.

## RESULTS

Available working time and the actual time spent per staff category at work were calculated giving due consideration to all the official leaves. The available working time was obtained by subtracting the duration of total absence from the total available working days in a year. Out of 52 weeks per year, 17 weeks are unavailable as working weeks, and the remaining 35 weeks are available as working time, which is 210 days or 1,680 hours per year. Government offices in Burdwan remain open for 8 hours on six working days in the entire year (Table 1).

Hospital statistics regarding different patient-care activities for all rural hospitals of Burdwan district is also given (Table 2). The activity standards that are set for nursing staff in an institution must be authoritative, i.e. acceptable to health professionals and to administrators. All the service components with standard time need to perform the work, i.e. activity standard set by senior nursing personnel was used in the calculation. Total workload in hours for an entire year was calculated applying activity standard and annual statistics for different patient-care services for indoor admitted patients, emergency, operation theatre, and labour room. Where statistics were available, workload was derived directly (Table 3 and 4). Where statistics were not available, recording and reporting allowances were derived from set activity standard (Table 5). At some places, proportion of patients for three months was taken from different department registers, and it was projected for the entire year for calculation. Then, from the total hours and AWT baseline staffing requirement, intermediate staffing requirement and calculated staffing requirement were derived for all the six rural hospitals. The WISN ratio was determined by dividing the present staffing in the hospitals with the calculated staffing requirements for each hospital in the district. For simplification of understanding, calculation for one (Manker) rural hospital has been shown in Table 6.

**Figure.**
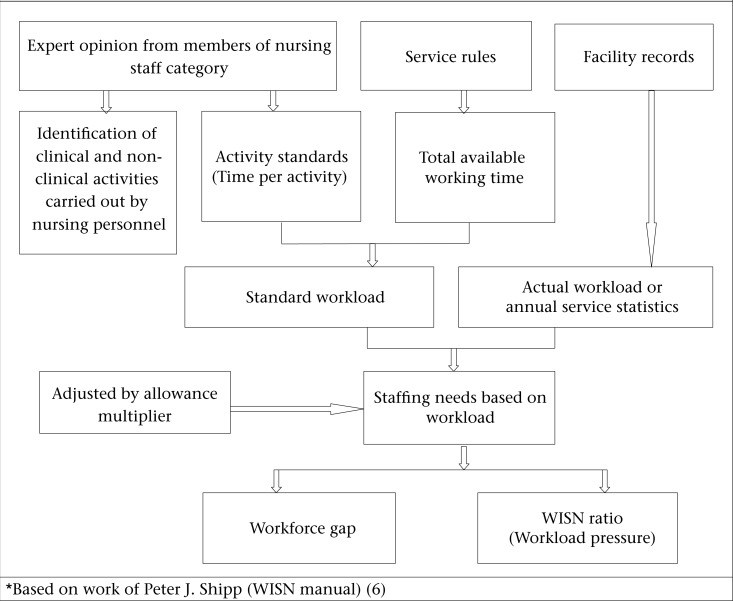
WISN methodology for calculating staffing needs^*^

**Table 1.** Annual working time calculation for nurses

Non-working days per year for nurses	
Annual leaves (casual leave + earned leave)=44 days (14+30)	
Sick leave	30 days
Holidays	28 days
Total	102 days (102/6=17 weeks/year)
Available working time* per year for nurses	
Working weeks = 52 − 17 = 35 per year Working days = 35 × 6 = 210 per year Working hours = 210 × 8 = 1,680 per year	

*Available working time is the amount of time available in a year, per staff category, for delivering health services

**Table 2.** Annual statistics in 2011 of all rural hospitals in Burdwan district

Name of rural hospital	Indoor admission	Patient referral	Death in indoor facilities	No. of OPD visits	No. of emergency cases	No. of tubectomy	No. of vasectomy	No. of LSCS	No. of normal deliveries	No. of livebirths	No. of stillbirths
Manker	2,960	448	48	64,890	11,654	174	Nil	38	619	614	5
Bhatar	6,011	1,100	19	110,393	14,150	281	Nil	Nil	958	955	3
Ballavpur	2,892	671	36	53,974	6,854	30	Nil	Nil	216	215	1
Singhat	1,402	145	6	73,542	11,287	396	186	Nil	514	508	6
Srirampur	1,928	1,114	7	56,099	12,696	10	Nil	Nil	421	421	0
Memari	6,007	1,802	66	122,066	12,625	331	Nil	Nil	1,078	1,065	13

For analysis, the staff members who were directly involved in different nursing care in hospital were considered; sister-in-charge was excluded from calculation. Rural hospital in Memari had maximum requirement of 32, in addition to the existing nursing staff working to bear the workload as shown by the last year's statistics and least requirement of 10 nurses shown by rural hospital in Ballavpur. Rural hospitals in Singhat, Srirampur, and Manker showed maximum workload pressure ranging from 70% to 75%. The WISN ratio for rural hospital in Manker turned out to be 0.30; for rural hospital in Singhat, it was only 0.25. It means there were only 25% staff members available as per their need; there was 75% deficit or 75% overburden (Table 7).

## DISCUSSION

As in many other developing countries, the Ministry of Health in India is constantly under pressure to provide better health services to an ever-increasing population and to maintain equity in the distribution of those services but resources are not keeping pace with the increase in demand. Thus, health administrators must make all efforts to achieve optimum utilization of resources, particularly human resources, in order to have greater impact (by improving current effectiveness levels) and equity in the provision of health services (i.e. overall staffing deployment according to demand) and economy of operation (by controlling staff categories, numbers, and mix). Population ratios and standard staffing schedules are generally used in determining the workforce requirements. These methods have serious limitations. Population ratios are based on all qualified staff in a particular category, irrespective of their specialty. On the other hand, with the standard staffing schedules, the distribution of the facilities themselves is a major factor determining staffing needs. None of these methods takes account of the wide local variations with respect to morbidity, access to facilities, etc. Staffing norms based on population ratios and standard staffing schedules are usually set somewhere in the middle of the range. This leads to overstaffing in some facilities and understaffing in others. The latter facilities, unable to cope with their workloads, apply for more staff and frequently get an increase because the request is, in fact, justified. Once this precedent has been established, other facilities also seek staff increases even though their staffing levels are in fact adequate for their workloads. Thus, the authority of the norms or standard staffing schedules disappears and their value in the management and control of human resources for health (HRH) is diminished. Using the WISN Method for human resource management in a district, by comparing the estimated results (ratios and differences) for a group of facilities, a manager can identify whether there are any staffing inequities between the facilities and, moreover, what can be done to improve the situation.

In our study, calculation as per WISN manual shows that there is a huge gap between number of current personnel working and calculated requirement as per WISN. A study undertaken in Bangladesh also indicates that health managers have a role in increasing efficiency and equity by making optional deployment of the workforce among health facilities (8). In one study done by Hagopian *et al*. (9) in Orissa state of India, they found, for 18 service centres of Ganjam district, that the health workforce supply should be enhanced by 43 additional physicians, 15 nurses, and 80 nurse-midwives, and these numbers probably also underestimate the need as they assume away geographic barriers; similar findings were obtained in our study also. In a study done in Africa by Musau *et al*. (10), using WISN, it was found that very few departments were optimally established, with the majority either under- or overstaffed. There were intra-departmental discrepancies in optimal levels of cadres even though many of them had the right number of total workforce in their teaching hospital.

**Table 3.** Ward-nurse activity standards for nursing staff of rural hospitals

Activity	Activity standard*
Admission and patient monitoring	1 hour per patient
Ward rounds and handing over	6 hours per day
Administering treatment	20 mins per patient
NGT feeding	30 mins per patient
Referral services	30 mins per patient
Bed-making	10 mins per patient
Turning patient	30 mins per patient
Death care	2 hours per death

*An activity standard is the time it would take for a well-trained and well-motivated member of a particular staff category to perform an activity at acceptable professional standards

The first use of the WISN Method in a country is usually to review the current staffing levels in relation to the current workloads in health facilities, using current medical procedures to current professional standards as described above. However, once the method is established, a whole range of further uses is then available to assist in planning health services and to help solve specific management problems as they arise.

### Limitations

Activity analysis can be undertaken with different methods, each with varying degrees of accuracy and cost. The WISN Method deliberately sets out to simplify the process. Still, the relative simplicity of the WISN Method makes it both appealing and understandable to those who must make judgements based on a WISN assessment.

**Table 4.** Selected patient-care activity standards for other nursing activities (beyond ward-nursing)

Activity	Activity standard
Outpatient clinic	
Patient counselling	15 minutes per patient
Injection administration	10 minutes per patient
Emergency (outside, beyond morning shift)	
Cases not admitted in indoor facility	30 minutes per patient
Operation theatre	
Pre-operative activities and circulating nursing activity	3 hours per patient
Post-operative care	30 minutes per patient
Assisting in minor operations, like tubectomy, vasectomy, etc.	30 minutes per patient
Pre-operative preparation and circulating nursing activity	30 minutes per patient
Post-operative care	30 minutes per patient
Labour room	
Mother care	2 hours per patient
Baby care	30 minutes per patient
Dead baby care	1 hour per death

The reliability of the WISN Method depends upon the accurate and extensive record-keeping and maintaining annual service statistics carefully. In the present study, we also faced difficulty in getting required information as it was not readily available (e.g. NGT feeding, turning patient); information on these was prospectively collected for the purpose of the study.

As all the rural hospitals of one district was chosen, the demographic situation and morbidity and mortality status, on one hand, and requirement of healthcare beneficiaries, on the other, are more or less similar everywhere; so, there was less chance of differences in services offered in the hospitals depending on local population health conditions or the types of medicine or surgery practised by the doctors or the physical facilities, availability of supplies, etc., which might create barriers to more efficient work.

**Table 5.** Category and individual allowance factor for nursing staff

Activity	Activity standard	Allowances
Cleaning and sterilizing equipment	1 hour per day	12.5% (CAS)
In-service training	6 days per year	2.85% (CAS)
Record-keeping and reporting	1 nurse, 4 hours per day	0.86 Whole time equivalent (WTE) (IAS)*
Others	1.5 hours per day	18.75% (CAS)**

*IAS=Individual allowances standard;

**CAS=Category allowances standard

**Table 6.** Distribution of WISN calculation for nursing staff of rural hospital in Manker

Total hours	32,525.5
Baseline staff requirement (total hours/available working time)	19.36
Allowance multiplier	1.51
Intermediate staffing requirement	29.23
Individual allowances standard	0.86 WTE
Calculated staffing requirement	30.09 Ω 30

This study was carried out among only one category of health personnel working in the facility. Precise estimation in the process of setting activity standard was not possible for all activities as there was possibility of sharing the activity by other category of workers as and when required. Moreover, estimation of activity standard may be affected by subjective method of setting the standard. The WISN Method overlooks the consequences of ‘errors’ arising from the invalid assumptions. The estimation by this method is based on current utilization rate of service and does not consider the possible increase in utilization rate in future.

### Conclusions and recommendations

The health sector of India is constantly under pressure to increase efficiency and equity. Appropriate human resources management and planning can contribute greatly to the improvement of efficiency and equity in the health sector. Presently, no objective criterion is followed in India in determining staffing needs as well as in allocating workforces. Deployment, transfer, posting, etc. are important issues for human resources management in the health sector. Workload indicator of staffing need (WISN) has been estimated for nurses working in different rural hospitals under Burdwan district of West Bengal. The average estimate of WISN turned out to be 0.35. However, there exists a wide variation of this figure among the rural hospital. It varies from 0.25 to 0.58. The presence of significant variation of WISN indicates that health managers have a role in increasing efficiency and equity by making optimal deployment of workforces among facilities. In a nutshell, planning for nursing personnel in Burdwan district can be done by using WISN Method for proper allocation and deployment so that workload can be evenly distributed among nursing staff in all hospitals to improve the healthcare services in rural hospitals.

**Table 7.** Staff analysis for hospitals using WISN Method

Name of hospital	Sanctioned posts (a)	Actual no. of posted staff, including sister in-charge (b)	No. of staff on deputation	Staff deficit/excess as per government norms	Staff involved in nursing care, excluding sister in-charge (c)	Calculated requirement (d) as per WISN	Differences (c-d)	WISN ratio (c/d)	Present staff as % of the required	Workload pressure (%)
Manker	12	10	0	-2	9	30	-21	0.3	30	70
Bhatar	18	18	0	0	17	47	-30	0.36	36	64
Srirampur	9	8	0	-1	7	25	-18	0.28	28	72
Singhat	17	8	0	-9	7	28	-21	0.25	25	75
Ballavpur	13	15	2	+2	14	24	-10	0.58	58	42
Memari	16	19	3	+3	18	50	-32	0.36	36	64

## ACKNOWLEDGEMENTS

The study was partially self-funded and partially funded by Indian Association of Preventive and Social Medicine (West Bengal Chapter), India, under grant for best research project proposal initiated by Prof. Akalanka Bhandari in 2012.

**Conflict of interest:** The authors declare no conflict of interest.
